# What is a tibial pilon fracture and how should they be acutely managed? A survey of consultant British Orthopaedic Foot and Ankle Society members and non-members

**DOI:** 10.1308/rcsann.2023.0049

**Published:** 2023-11-01

**Authors:** DS Hill, JR Davis, on behalf of the ENFORCE collaborative

**Affiliations:** Torbay and South Devon NHS Foundation Trust, UK

**Keywords:** Pilon, Fracture, Definition, Management

## Abstract

**Introduction:**

Controversy exists around which distal tibial fractures are pilon fractures. We evaluated views to define a pilon fracture and support the development of standards of care.

**Methods:**

Views regarding the characteristics of a pilon fracture and acute soft tissue management were determined through a questionnaire. This was trialled, approved by the British Orthopaedic Foot and Ankle Society and distributed to its members. This was also distributed nationally as part of the ENFORCE study.

**Results:**

In total, 282 consultants from 27 units responded, of whom 24% (69/282) were foot and ankle specialists. Some 58% (163/282) agreed that a pilon fracture is primarily a soft tissue injury, 81% (228/282) that pilon fractures occur though high-energy transfer, 81% (228/282) that pilon fractures are sustained through an axial compression mechanism and 93% (265/282) that they are a potentially limb-threatening injury. Overall, 83% (234/282) agreed that in a length-unstable pilon fracture it is not possible to maintain the talus near anatomically under the tibial plafond without rigid fixation to control length – with 87% (246/282) agreeing that the acute first-line management should be a spanning external fixator. Opinions were that the time frame between diagnosis and intervention should be: less than 6h (63%; 154/246), 6–12h (31%; 77/246) and 12–24h (6%; 15/246).

**Conclusion:**

Consensus supports defining a pilon fracture as a potentially limb-threatening high-energy axial compression injury, and a spanning external fixator as the first-line management of a length-unstable injury less than 12h from diagnosis.

## Introduction

‘Pilon’, the French word for pestle, was first used by Etienne Destot in 1911 as an analogy for the mechanism of injury from the talus on the distal tibia.^1^ The term ‘pilon fracture’ is commonly used to describe bony injury to the tibial plafond resulting from axial compression of the talus into the articular surface of the distal tibia. These fractures are difficult to treat, potentially limb-threatening and have a poor prognosis.^2^ Controversy exists around which intra-articular fractures of the tibial plafond are considered true ‘pilon’ fractures,^1^ and how they should be managed, both acutely and definitively.^3^ Although no published consensus exists, descriptive features have included (Table 1): high-energy axial loading of the talus into the tibial plafond,^7^ exploding the articular surface^4^ with severe soft tissue damage.^8^ Evidence to inform the management of tibial pilon fractures is lacking.^5^ Our aim was to evaluate views from both foot and ankle specialists, and other specialty consultant orthopaedic surgeons, to define a tibial pilon fracture and how they should be managed acutely.

**Table 1:** Definitions used to describe a ‘pilon fracture’ in the literature

High-energy tibial ‘pilon’ fractures are due to axial loading with the talus driven into the distal tibia, exploding the distal tibial articular surface with impaction of the comminuted metaphyseal bone, and with occasional proximal diaphyseal extensions.^2^
Pilon fractures are distal tibia end fractures affecting the weight-bearing articular surface.^3^
A pilon fracture of the tibia involves the horizontal articular surface of the distal tibia with proximal extension. The injury is usually caused by a high-energy axial force which also produces severe soft tissue damage.^4^
A pilon fracture is a severe fracture of the distal end of the tibia, involving its weight-bearing articular surface at the ankle joint.^5^
These fractures are caused by axial loading in which the talus is driven into the plafond, resulting in articular impaction of the distal tibia.^6^

## Methods

### Design

We designed a cross-sectional survey to canvas the opinions of consultant orthopaedic surgeons. This was trialled across five South West Peninsula hospitals in the United Kingdom and refined. The final version was reviewed and approved by the British Orthopaedic Foot and Ankle Society (BOFAS) scientific committee (Table 2) who sponsored the survey, and distributed to its membership. The same survey was also distributed to consultants in the 27 hospitals partaking in the ENFORCE study, a trainee-led multicentre observational study of the acute management of tibial pilon fractures.

**Table 2:** Consultants reported views on the defining features of a tibial Pilon fracture

** **	**Foot and Ankle Subspecialtyconsultants** **(*n*=69)**	**Non Foot and Ankle Subspecialtyconsultants ** **(*n*=213)**	**All responses** **(*n*=282)**
**A Pilon fracture is primarily a soft tissue injury?**			
Strongly agree Agree Neutral Disagree Strongly disagree	19% (13/69) 39% (27/69) 28% (19/69) 12% (8/69) 3% (2/69)	21% (45/213) 37% (78/213) 27% (57/213) 15% (31/213) 1% (2/213)	21% (58/282) 37% (105/282) 27% (76/282) 14% (39/282) 1% (4/282)
**A Pilon fracture is sustained though a high energy transfer mechanism (i.e. fall form > 2m, RTA, industrial accident)?**			
Strongly agree Agree Neutral Disagree Strongly disagree	20% (14/69) 57% (39/69) 17% (12/69) 16% (4/69) 0% (0/69)	24% (51/213) 58% (124/213) 14% (30/213) 4% (8/213) 0% (0/213)	23% (65/282) 58% (163/282) 15% (42/282) 4% (12/282) 0% (0/282)
**A Pilon fracture is sustained from an axial compression mechanism?**			
Strongly agree Agree Neutral Disagree Disagree Strongly disagree	26% (18/69) 33% (23/69) 33% (23/69) 7% (5/69) 0% (0/69)	22% (47/213) 66% (140/213) 9% (19/213) 3% (7/213) 0% (0/213)	23% (65/282) 58% (163/282) 15% (42/282) 4% (12/282) 0% (0/282)
**All intra-articular fractures of the tibial plafond be classified as a Pilon fracture?** ** **			
Strongly agree Agree Neutral Disagree Strongly disagree	4% (3/69) 30% (21/69) 16% (11/69) 38% (26/69) 12% (8/69)	3% (7/213) 32% (69/213) 17% (35/213) 40% (86/213 8 % (16/213)	4% (10/282) 32% (90/282) 16% (46/282) 40% (112/282) 9% (24/282)
**Pilon fracture can be sustained from a fall from standing height?** ** **			
Strongly agree Agree Neutral Disagree Strongly disagree	6% (4/69) 57% (39/69) 20% (14/69) 17% (12/69) 0% (0/69)	4% (8/213) 60% (127/213) 19% (40/213) 18% (38/213) 0% (0/213)	4% (12/282) 59% (166/282) 19% (54/282 18% (50/282) 0% (0/282)
**A length unstable Pilon fracture refers to the situation where it is not possible to reduce and maintain the talus near anatomically under the tibial Plafond without rigid fixation to control length (i.e. an external fixator or open reduction internal fixation)?** ** **			
Strongly agree Agree Neutral Disagree Strongly disagree	22% (15/69) 65% (45/69) 12% (8/69) 1% (1/69) 0% (0/69)	15% (31/213) 71% (152/213) 11% (24/213) 3% (6/213) 0% (0/213)	16% (46/282) 70% (197/282) 11% (32/282) 2% (7/282) 0% (0/282)
**A distal tibial Pilon fracture is a potentially limb threatening injury?** ** **			
Strongly agree Agree Neutral Disagree Strongly disagree	45% (31/69) 52% (36/69) 3% (2/69) 0% (0/69) 0% (0/69)	40% (86/213) 53 % (112/213) 4% (9/213) 3 % (6/213) 0% (0/213)	41% (117/282) 52% (148/282) 4% (11/282) 2% (6/282) 0% (0/282)

### Inclusion criteria

Our survey was distributed to all BOFAS consultant members (*N* = 608), and all consultants working in the 27 collaborating units of the ENFORCE study (total denominator unknown). Respondents were asked to confirm that they were practising consultant orthopaedic surgeons responsible for the acute (first 6h) management of tibial pilon fractures. To minimise the chances of duplicate responses we requested that respondents did not complete the survey distributed by our ENFORCE study collaborators if they had already completed the BOFAS survey.

### Distribution

The BOFAS webmaster distributed our survey to its membership using Survey Monkey. ENFORCE study collaborators distributed the same survey questions via a separate Survey Monkey link. Both were followed up with two separate reminders.

### Analysis

Responses from both BOFAS consultant members and ENFORCE study consultant collaborators were combined into a single database. Analysis was performed for all responses collectively, but also split by those reporting a subspecialty interest in foot and ankle surgery, and the remaining responders.

## Results

Some 282 consultant orthopaedic surgeons, from 27 units, with responsibility for the acute management of tibial pilon fractures responded. These consisted of 61 BOFAS consultant members (10% response rate: 61/608) and 221 ENFORCE study consultant collaborators (unable to report response rate). In all, 24% (69/282) declared a subspecialty practice in foot and ankle surgery. Respondents reported their number of years practising as a consultant as: mean 9 years, median 8 years and range 1–28 years.

Views on the defining features of a tibial pilon fracture are given in Table 2. Some 58% of respondents (163/282) agreed that a pilon fracture is primarily a soft tissue injury; 81% (228/282) agreed that pilon fractures occur though a high-energy transfer; 81% (228/282) agreed that a pilon fracture is sustained through an axial compression mechanism; 36% (100/282) agreed that all intra-articular fractures of the tibial plafond be classified as a pilon fracture; 63% (178/282) agreed that a pilon fracture can be sustained from a fall from standing height; 86% (243/282) agreed that a length-unstable pilon fracture refers to the situation where it is not possible to reduce and maintain the talus near anatomically under the tibial plafond without rigid fixation to control length (i.e. an external fixator or open reduction internal fixation); and 93% (265/282) agreed that a pilon fracture is a potentially limb-threatening injury.

Reported views on the acute management of tibial pilon fractures are detailed in Table 3. In total, 68% (192/282) reported that the acute management of a length-stable tibial pilon fracture to achieve soft tissue damage control should be closed reduction and application of a plaster cast, with the ankle joint remaining reduced. In the context of achieving acute soft tissue damage control in a length-unstable pilon fracture, 87% (245/282) reported that this should be with a spanning external fixator. Some 61% (174/282) of respondents stated that acute damage control of the soft tissues should be achieved within 6h from primary radiological diagnosis. In total, 63% (176/282) agreed that intervention to achieve soft tissue damage control in a distal tibial pilon fracture is an indication for rapid sequence induction anaesthesia if a patient is not appropriately starved. Some 67% (190/282) agreed that joint reduction to achieve soft tissue damage control in distal tibial pilon fractures must be radiographically confirmed and documented before transfer from the emergency department, and if this is not possible then the patient should be transferred directly to the operating theatre, with 64% (179/282) supporting this being provided 24h a day.

**Table 3:** Consultants reported views on the acute management of tibial Pilon fractures

	**Foot and Ankle Subspeciality consultants (n=69)**	**Non Foot and Ankle Subspeciality consultants (n=213)**	**All responses (n=282)**
**The first line acute management of a LENGTH STABLE distal tibial Pilon fracture to achieve soft tissue damage control should be…?**			
Application of a plaster cast in situ Closed reduction and application of a plaster cast, and the ankle joint remains reduced Closed reduction and application of a spanning external fixator Primary definitive fixation by the on-call team	0% (0/69) 68% (47/69) 28% (19/69) 4% (3/69)	8% (18/213) 68% (145/213 22% (47/213) 1% (3/213)	6% (18/282) 68% (192/282) 23% (66/282) 2% (6/282)
**The first line acute management of a LENGTH UNSTABLE distal tibial Pilon fracture to achieve soft tissue damage control should be…?**			
Application of a plaster cast in situ Closed reduction and application of a plaster cast, and the ankle joint remains reduced Closed reduction and application of a spanning external fixator Primary definitive fixation by the on-call team	0% (0/69) 19% (13/69) 81% (56/69) 0% (0/69)	0% (0/213) 11% (24/213) 89% (189/213) 0% (0/282)	0% (0/282) 13% (37/282) 87% (245/282) 0% (0/282)
**In the context of your choice above soft tissue damage control should be achieved and radiographically documented within ….?**			
Less than 6 hours from radiological diagnosis Between 6-12 hours from radiological diagnosis Between 12-24 hours from radiological diagnosis Greater than 24 hours from radiological diagnosis	94% (65/69) 3% (2/69) 3% (2/69) 0% (0/69)	52% (109/282) 38% (82/282) 10% (22/282) 0% (0/282)	61% (174/282) 30% (84/282) 9% (24/282) 0% (0/282)
**Do you think that intervention to achieve soft tissue damage control in a distal tibial Pilon fracture is an indication for rapid sequence induction anaesthesia if a patient is not appropriately starved (i.e starving status should not delay intervention)?**			
Strongly agree Agree Neutral Disagree Strongly disagree	9% (6/69) 80% (55/69) 4% (3/69) 7% (5/69) 0% (0/69)	5% (10/213) 49% (105/213) 23% (49/213) 21% (44/213 2% (5/213)	6% (16/282) 57% (160/282) 18% (52/282) 17% (49/282) 2% (5/282)
**Do you feel that joint reduction to achieve soft tissue damage control in distal tibial Pilon fractures must be radiographically confirmed and documented before transfer from the Emergency Department, and if this is not possible then the patient should be transferred directly to the operating theatre?**			
Strongly agree Agree Neutral Disagree Strongly disagree	17% (12/69) 59% (41/69) 2% (2/69) 20% (14/69) 0% (0/69)	14% (26/213) 45% (84/213) 14% (26/213) 24% (45/213) 3% (6/213)	13% (38/282) 54% (152/282) 10% (28/282 21% (59/282) 2% (6/282)
**Should this service be provided 24 hours a day?**			
Strongly agree Agree Neutral Disagree Strongly disagree	26% (18/69) 57% (39/69) 9% (6/69) 9% (6/69) 0% (0/69)	13% (27/213) 45% (95/213) 19% (39/213) 20% (42/213) 5% (10/213)	16% (45/282) 48% (134/282) 16% (45/282) 17% (48/282) 4% (10/282)

## Discussion

Despite the severity, high complication rate and poor functional outcomes associated with tibial pilon fractures, there is a lack of clarity in the literature around the definition of a pilon fracture, together with an absence of standards of care. Our survey highlights support for a number of defining characteristics of a tibial pilon fracture and how they should be managed.

### Definitions

We found varying levels of agreement between respondents for each of the proposed defining criteria of a tibial pilon fracture (Table 2). There was no notable difference between foot and ankle specialists and other specialty consultants in any of the themes, apart from there being proportionately less support from the foot and ankle subspecialists for the statement relating to pilon fractures being sustained though an axial compression mechanism. We suspect this is a recognition from specialists leading definitive management that the concept of a rotational mechanism pilon fracture exists.^9^ Although not prescriptive in terms of fracture types and patterns (e.g. posterior malleolus vs posterior pilon fracture), our definition does describe a pilon fracture as a potentially limb-threatening injury and we hope this will provide focus for their acute management Table 4.

**Table 4:** Proposed definition of a tibial pilon fracture

**Pilon fracture:**
“*A Pilon fracture is a potentially limb threating injury of the tibial plafond joint surface and surrounding soft tissues, typically sustained though an axial compression high energy mechanism, recognising that similar levels of destruction can be sustained from lower energy mechanisms in certain patient groups, and that not all intra-articular fractures of the tibial plafond are Pilon fractures.*”
**Length STABLE Pilon fracture:**
“*A length stable Pilon fracture refers to the situation where it is possible to reduce and maintain the talus near anatomically under the tibial Plafond without rigid fixation to control length”* (NB: it is not possible to achieve this in complete articular injuries).
**Length UNSTABLE Pilon fracture:**
“*A length unstable Pilon fracture refers to the situation where it is not possible to reduce and maintain the talus near anatomically under the tibial Plafond without rigid fixation to control length*” (NB: both partial and complete articular injuries can be length unstable).

### Standards of care

**Figure 1:**
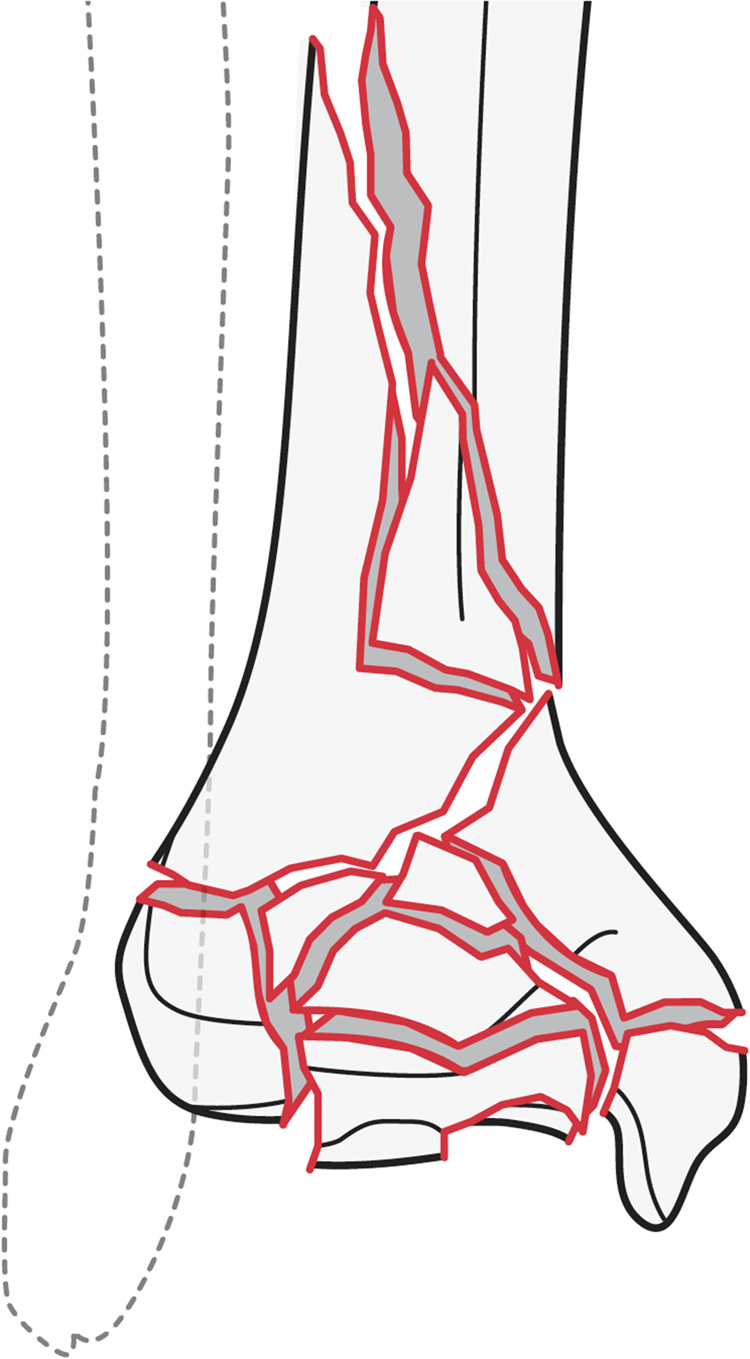
The acute management of tibial pilon fractures

An ankle fracture British Orthopaedic Association Standards for Trauma (BOAST) endorsed by BOFAS was published in 2016, but the same has not been produced for tibial pilon fractures. Given the complexity of managing tibial pilon fractures, the high incidence of complications and the associated costs of managing these complications, the need for subspecialty management guidance is clear. We propose standards of care for the acute soft tissue damage control (Figure 1 and Table 5), which could contribute towards a BOAST for the management of tibial pilon fractures.

**Table 5:** Proposed standard of care for the acute management of tibial pilon fractures

The first line management of a tibial Pilon fracture is to achieve soft tissue damage control.
In length stable injuries this should be achieved with a closed reduction and application of a plaster cast, with the ankle joint remaining reduced and radiologically documented within 6 hours of primary radiological diagnosis.
In length unstable injuries this should be achieved with spanning external fixator within 6 hours of primary radiological diagnosis.
Joint reduction must be radiographically confirmed before transfer from the Emergency Department. If this is not possible then the patient should be transferred directly to the operating theatre for intervention to achieve soft tissue damage control. Provision for this must exist 24 hours a day.
Intervention to achieve soft tissue damage control in a tibial Pilon fracture is an indication for rapid sequence induction anaesthesia if a patient is not appropriately starved.

We found little difference between foot and ankle specialists and other specialty consultants in the indicated first-line management of both length-stable and length-unstable injuries with regards to achieving soft tissue damage control. A notable difference was seen in terms of the time frame within which respondents felt this should be achieved and radiologically documented. Some 94% (65/69) of foot and ankle specialists stated that soft tissue damage control should be achieved and radiologically documented within 6h from primary radiological diagnosis, whereas the same was reported by only 52% (109/282) of non-foot and ankle specialist consultants, with a further 38% (82/282) stating that it would be acceptable for this to be achieved within 12h. This difference likely reflects the fact that it is the foot and ankle specialists who definitively management these injuries and their complications. Similarly, a difference between foot and ankle specialists and other specialty consultants was reported for the levels of agreement with defining intervention to achieve soft tissue damage control in a tibial pilon fracture as an indication for rapid sequence induction anaesthesia if a patient is not appropriately starved (i.e. starving status should not delay intervention). Some 89% (61/69) of foot and ankle specialists supported this proposed standard of care, compared with only 54% (115/213) of other specialty consultants. The National Confidential Enquiry into Patient Outcome and Death (NCEPOD)^10^ classification of surgical interventions defines the urgency with which surgery should be performed. Immediate intervention is indicated for life- or limb-saving interventions (e.g. fracture with a major neurovascular deficit or compartment syndrome) and surgery should be commenced within minutes of the decision to operate. Urgent intervention is indicated for potentially life- or limb-threatening conditions (e.g. critical organ or limb ischaemia) and surgery should be commenced within hours of the decision to operate. Achieving acute soft tissue damage control in a length-unstable pilon fracture should be considered an ‘urgent’ indication for taking a patient to the emergency theatre. The NCEPOD classification for urgent intervention within “hours” is very vague (e.g. 1–23h), and there is a need for a more specific time frame to give surgeons support in managing these potentially limb-threatening injuries in a timely manner. Our experience is that many fail to recognise a length-unstable pilon fracture (or indeed a grossly dislocated ankle fracture with compromised skin) as an urgent indication for emergency theatre. A significant reluctance to open additional theatres to accommodate such patients occurs if emergency theatres are already occupied with often lengthy cases from other specialties.

## Conclusion

Given the heterogeneity of patients, mechanisms of injury and resultant damage to soft tissues and bone, a clear panacea to guide the management of these complex injuries is a long way off. We argue that a standard of care for tibial pilon fractures is mandated to increase the profile and standardise acute management of these potentially limb-threatening injuries, together with setting them apart from more straightforward ankle fractures.
